# Breast Cancer Screening in Malaysia: A Policy Review

**DOI:** 10.31557/APJCP.2021.22.6.1685

**Published:** 2021-06

**Authors:** Mila Nu Nu Htay, Michael Donnelly, Desiree Schliemann, Siew Yim Loh, Maznah Dahlui, Saunthari Somasundaram, Nor Saleha Binti Ibrahim Tamin, Tin Tin Su

**Affiliations:** 1 *Centre for Population Health (CePH), Department of Social and Preventive Medicine, Faculty of Medicine, University of Malaya, Kuala Lumpur, Malaysiaa. *; 2 *Department of Community Medicine, Melaka-Manipal Medical College, Manipal Academy of Higher Education (MAHE), Melaka, Malaysia. *; 3 *Centre for Public Health and UKCRC Centre of Excellence for Public Health, Queen’s University Belfast, Belfast, UK. *; 4 *Department of Rehabilitation Medicine, University of Malaya, Malaysia. *; 5 *Department of Health Policy and Management, Faculty of Public Health, Universitas Airlangga, Surabaya, Indonesia. *; 6 *National Cancer Society, Kuala Lumpur, Malaysia.*; 7 *Ministry of Health, Putrajaya, Malaysia. *; 8 *South East Asia Community Observatory (SEACO), Monash University Malaysia, Bandar Sunway, Malaysia. *

**Keywords:** Breast cancer, screening, policy review, breast cancer early diagnosis, Malaysia

## Abstract

**Background:**

Breast cancer is the leading cause of cancer among Malaysian women. The implementation of prevention measures including screening has the potential to reduce the burden of breast cancer which caused by late presentation.

**Aims:**

This paper aimed to review the public health policy relating to breast cancer screening in Malaysia that was undertaken in order to contribute to policy development regarding cancer prevention, detection and the improvement of services for Malaysian women.

**Methods:**

The policy review strategy included a specific search of the website of the Ministry of Health in Malaysia for relevant policies. In addition, we searched Google and Pubmed for breast cancer screening programmes, policies, and guidelines for women in Malaysia. In addition, experts and stakeholders provided additional resources, published in Malay language. Relevant guidelines in the Malay language were translated into English and included the document review.

**Results:**

The policy analysis indicated that although it is known that screening, early detection and diagnosis improve survival rates, delayed diagnosis remains a significant issue. The Ministry of Health policy stipulates the provision of opportunistic mammography screening. However, the uptake is varied, and implementation is challenging due to a lack of awareness about screening and difficulties related to accessing services, especially in rural areas. The establishment and implementation of referral guidelines is essential to receive timely treatment for breast cancer patients. There is a need to enhance the cancer reporting by the doctors to the national cancer registry, in collaboration with government services and the private cancer-care sector to improve the monitoring and evaluation of cancer control policies and programmes.

**Conclusion:**

A focus on raising awareness, increasing the accessibility of screening facilities and improving referral processes and the overall connectivity of the cancer care system are key steps to down-staging breast cancer in Malaysia.

## Introduction

Breast cancer (BC) is a global health threat and the leading cause of cancer among women impacting approximately two million women each year (WHO, 2018). The growing burden of cancer also affects women in Asia, including Malaysia, where the age standardised incidence rate (ASR) is 34.1 per 100,000 population (Ministry of Health Malaysia, 2019b). Breast cancer incidence in Malaysian multi-ethnic society varies from 1 in 22 Chinese women followed by 1 in 23 Indian women and 1 in 30 Malay women (Ministry of Health Malaysia, 2019b). Women who develop breast cancer have an 81% (Stage II) to 88% (Stage I) chance of 5-year survival if their cancer is diagnosed early whereas it is much lower for diagnosed cancer at Stage III (60%) or IV (23%) (NationalCancerRegistry, 2018). The implementation of prevention measures including screening has the potential to reduce the burden of breast cancer which caused by late presentation (Al-Amri, 2005; Njor et al., 2012; Massat et al., 2016). However, approximately 48% of breast cancer cases in Malaysia are diagnosed late ( Ministry of Health Malaysia, 2019b). 

This paper presents the results of a review of public health policy relating to breast cancer screening in Malaysia that was undertaken in order to contribute to policy development regarding cancer prevention, detection and the improvement of services for Malaysian women. 

The policy review was part of a larger collaborative Malaysia-UK global health study designed to develop and evaluate a cancer awareness-raising programme for Malaysia. The policy appraisal process comprised iterative discussions and document review between members of the collaboration. Representation included the Ministry of Health in Malaysia (a public health doctor with responsibility for cancer policy), the National Cancer Society in Malaysia (the Director of the lead patient advocacy organisation), public health, primary care and rehabilitation specialists with expertise in cancer prevention (from the University of Malaya and Monash University, Malaysia), community medicine (from Melaka-Manipal Medical College, Malaysia) and public health at Queen’s University Belfast.

*Methods*

The policy review strategy included a specific search of the website of the Ministry of Health in Malaysia for relevant policies. In addition, we searched Google and Pubmed for breast cancer screening programmes, policies, and guidelines for women in Malaysia. Keywords included (breast cancer OR breast carcinoma) AND (policy OR strategy OR guideline) AND Malaysia. In addition, experts and stakeholders provided additional resources, published in Malay language. Relevant guidelines in the Malay language were translated into English and included the document review. A total of 126 articles and documents were identified, of which eight clinical guidelines, policy, and strategy documents for breast cancer screening in Malaysia were selected for review and documentary analysis. 

MNNH led the documentary analysis - reading the relevant documents, charting a policy development timeline, extracting policy goals and commitments from each document, comparing policy statements, plans and commitments in later dated documents with earlier documents, examining and noting congruence and discord between documents and discussing and debating these results and agreeing conclusions within the context of our multidisciplinary, multi-sectoral research team. Finally, our documentary analysis and subsequent discussions and reporting were informed by the results of the small number (to date) of published research papers about breast cancer in Malaysia, international evidence (eg Cochrane reviews) and seminal reports from thought leaders, principally the World Health Organization (WHO).

*Breast cancer screening guidelines and policies in Malaysia*

Malaysia’s healthcare system comprises public (government-funded) and private healthcare services (Quek, 2014). Clinical breast examinations (CBE) are performed in each sector. Opportunistic mammography screening is offered at government clinics (with mammogram facilities), private hospitals and recently a subsidized mammogram programme was implemented at private healthcare facilities designated by the National Population and Family Development Board (NPFDB) Malaysia (Mahmud and Aljunid, 2018). 

*Breast cancer screening guidelines in Malaysia*

*Clinical Practice Guidelines (CPG 1st and 2nd editions) for the management of Breast Cancer*

A CPG was produced for the first time in 2002 (MinistryofHealthMalaysia, 2002). Recommendations for mammogram use focused on women at high-risk of developing breast cancer, that is, women with “…past history of breast cancer and/or ovarian cancer, having family history of breast cancer, and a history of atypica on previous breast biopsy below 40 years” (Ministry of Health Malaysia, 2002). A further recommendation was that the value of mammograms should be explained to asymptomatic high-risk women who should receive ‘annual screening with a mammogram (if aged 40-49 years) and annually or biennially screening (if aged 50-75 years) (Ministry of Health Malaysia, 2002). 

The second CPG (updated in 2010) recommended mammography screening for the general population of women aged 50-74 years; and that MRI screening should be offered to high-risk women with a history of invasive breast cancer, ionising radiation exposure from breast cancer treatment, carriers of BRCA1 and 2 gene, a first degree family history of breast cancer and diseases such as Hodgkin’s disease (Ministry of Health Malaysia, 2010a). Table 1 presents the CPG (2010) recommendations differentiated according to the general population and the high-risk population. The CPG (2010) recommended (for the first time) that the general population of women should perform breast self-examination (BSE in order improve awareness about unusual breast changes (Ministry of Health Malaysia, 2010a) as well as continuing to recommend opportunistic screening with biennial mammograms for women aged 50-74 years (Ministry of Health Malaysia, 2010a). It was not recommended that women aged 40-49 years with low and intermediate risk should receive a mammogram routinely though it was stated that they should not be denied one if they express a wish to undergo mammography screening (Ministry of Health Malaysia, 2010a). Furthermore, it was recommended that breast cancer screening should commence at 30 years-old for high-risk women (Table 1); and that combined mammogram and MRI screening should be offered annually to improve the likelihood of detecting breast lesions (Ministry of Health Malaysia, 2010a) with the caveat that ‘MRI screenings should not be performed in patients with lobular carcinoma in situ and atypical hyperplasia’ (Ministry of Health Malaysia, 2010a) – see Table 1. 

*Garis Panduan Program Pengesanan Awal Kanser Payudara Kebangsaan - National Breast Cancer Screening Programme Guidelines, 2011*

In 2011, the national breast cancer screening guidelines was published by Family Health Development Unit, Ministry of Health (MoH) Malaysia (Ministry of Health Malaysia, 2011). The guideline recommended opportunistic CBE screening once every three years for women aged between 20 to 39 years; and annually for women aged 40 years and above. Women at high-risk were expected to undergo CBE annually, regardless of their age (Ministry of Health Malaysia, 2011). Furthermore, women aged 40 years and above were recommended to undergo mammography screening annually if at least one criterion under Criteria A in Table 1 was met and biennially if at least two criteria under Criteria B were met (see Table 1). 

*Clinical Practice Guidelines (CPG, 3rd edition) for the management of breast cancer*

The third edition of CPG for breast cancer (Ministry of Health Malaysia, 2019a) provided risk categorization groups that, for example, classified a lifetime risk of 17%-30% as moderate and 30% or more as high-risk. (Ministry of Health Malaysia, 2019a) based on an adaptation of the, National Institute for Health and Clinical Excellence. Familial breast cancer: classification, care and managing breast cancer and related risks in people with a family history of breast cancer (NICE guidelines, 2018). CBE is recommended for women 35 years and above as the risk of breast cancer is higher after this age in Malaysia. Biennial mammogram is recommended for the general population of women aged 50-74 years; annually for women in the moderate risk group (40-49 years old), annually or biennial between 50-59 years, and biennial from 60 years onwards (Ministry of Health Malaysia, 2019a). ‘Intensive screening’ is recommended for high-risk women and genetic carriers of BRCA using CBE, mammogram and MRI screening modalities. Regarding the high-risk category without genetic variants, mammogram should be considered for women, 30-39 years, an annual mammogram offered to 40-59 year olds and biennially from 60 years onwards. Women in high-risk group with BRCA1, BRCA2 and PALB2 should be offered an annual MRI between 30-49 years, annual mammogram between 40-69 years, and biennial mammogram from 70 years onwards (Ministry of Health Malaysia, 2019a). See Table 1. In addition, early referral within two weeks is recommended for “women aged 35 years and above with breast cancer signs and symptoms, high-risk women with signs and symptoms and patients with clinical signs of malignancy” (Ministry of Health Malaysia, 2019a). 

*National policies and strategies for the prevention and control of cancer*

Summaries of the key points in the guidelines and strategic plans/ policies are presented in Table 2. 

*National Strategic Plan for Non-Communicable Diseases (NSP-NCD)*

The first National Strategic Plan for Non-Communicable Disease (2010- 2014) which was published in 2010 focused mainly on the prevention and control of cardiovascular diseases and diabetes (Ministry of Health Malaysia, 2010b) and did not include criteria for monitoring BC screening services. A behavioural surveillance survey (to assess diet, physical activity and health-related outcomes) and a NCD risk factor surveillance survey were used biennially to monitor the NSP-NCD (Ministry of Health Malaysia, 2010b). These ‘outcome indicators’ suggested that progress was uneven during the 4-year implementation period and there was a lack of specific indicators and evaluation criteria for aspects of the strategy as well as limited funding (Ministry of Health Malaysia, 2016b). 

It was not until the second and current National Strategic Plan for Non-Communicable Disease (2016- 2025) was published that the prevention and control of cancer in Malaysia was included though there was no specific reference to BC screening (MinistryofHealthMalaysia, 2016b). The second NSP-NCD (2016-2025) comprised seven indicators with specific targets including the target to reduce ‘risk of premature mortality from cardiovascular diseases, cancer, diabetes, or chronic respiratory diseases’ from 20% (baseline) to 15% by 2025 (Ministry of Health Malaysia, 2016b).

*National Cancer Control Blueprint (2008-2015)*

The National Cancer Control Blueprint (NCCB, 2008-2015) by MoH included seven policy goals designed to reduce cancer burden in Malaysia. Goal 2 focused on Screening and Early Detection with specific objectives to ‘detect potentially cancerous lesions in the population at risk for the selected cancers [i.e. breast cancer, cervical cancer, oral cancer, liver cancer, colorectal cancer, prostate cancer and nasopharyngeal cancer]’ and to ‘increase the detection rate of selected cancers at an earlier stage’. The NCCB specified the achievement of goals by 2015 including a goal to offer mammograms systematically to the population. Mammography screening for high-risk women was initiated during the implementing period. However, mammography was used as diagnostic tool only and community or population-based screening remained out of reach (Ministry of Health Malaysia, 2008a). Whilst the practice of BSE was encouraged, more attention was directed at CBE – every woman (aged 35-49 years) who attended public health (PH) clinics (2008-2010) and 80% of women (aged 35-49 years) who attended PH clinics (2011 and 2015) were expected to receive a CBE. However, details about the achievement of targets were not reported.

*National Strategic Plan for Cancer Control Program (NSP-CCP) 2016-2020*

In 2015, the NCCB (2008-2015) was reviewed by a MoH-coordinated team of oncologists, clinicians, public health specialists and policy makers. The review led to the publication of the National Strategic Plan for Cancer Control Program (NSP-CCP) 2016-2020. NSP-CCP focused on nine different areas of cancer control including primary prevention, screening, palliative care, traditional medicine, and research on cancer and listed specific indicators for the evaluation of the policy (Ministry of Health Malaysia, 2017). Regarding breast cancer screening, CBE, mammogram and MRI (for high-risk women) are offering for early detection; which aimed to increase detection rate of early stages (stage 1 and 2) from 57% in 2011 to 60% by 2020. The monitoring and evaluation of the NSP-CCP including the cancer screening programme, development of a cancer screening monitoring system and a quality assurance procedure such as the auditing of mammogram centres were to be implemented between 2016 and 2020 by the Ministry of Health in collaboration with the Ministry of Education, non-governmental organisations and the College of Radiology (Ministry of Health Malaysia, 2017). 

The initiation of mammography screening among high-risk women in 2012 was an achievement of the NCCB policy (2008-2015). In line with policy direction, clinical guidelines updated the recommendation of targeting screening at high-risk women to screening the general population opportunistically via use of CBE and mammograms (Ministry of Health Malaysia, 2002; Ministry of Health Malaysia, 2011). CBE uptake increased from of 52% to 65% and mammogram uptake increased from 8% to 24% between 2006 to 2014 (Ministry of Health Malaysia, 2008b; LPPKN, 2017). However, late stage diagnosis was still common and, indeed, the most recent cancer registry report indicated an increase in 2016 (48%) compared to 2011 (43%) (Ministry of Health Malaysia, 2016a; Ministry of Health Malaysia, 2019b).

*Evaluation of cancer policies and strategies*

Rigorous evaluation of cancer control policies and strategies are crucial to monitor progress, learn lessons from implementation and guide future policy development (Alberg et al., 2013). Multiple policy documents have been published in Malaysia designed to implement cancer control as an integrated approach. However, formal policy evaluation studies appear to be sparse. This paper presents the first systematic policy examination.

*Implementation challenges*

The current mammography screening programme in Malaysia employs an opportunistic screening strategy (Ministry of Health Malaysia, 2011). A WHO position paper (2014) about the implementation of screening programmes in low resource settings concluded that the cost-effectiveness and implementation feasibility of population-based mammography screening was unclear and that CBE remained an important measure of early detection (WHO, 2014). Population-based mammography screening biennially was recommend only for women aged 50-69 years and only if key criteria were met (WHO, 2014). It was not recommended for women under 50 years or women who were >70 years old. WHO recommended also that the resource limited setting had to have a relatively strong healthcare system (WHO, 2014). The current Malaysian healthcare system has a number of challenges to overcome in order to meet implementation criteria. For example, a subsidized programme has been offered to eligible women in order to improve the affordability of mammography screening. However, a pilot cost-effectiveness evaluation of the programme at a private hospital in Selangor State (Lee et al., 2017) was inconclusive and the implementation of population-based screening remains a challenge in terms of resources and cost-effectiveness in Malaysia. 

CBE and mammography screening strategies (as described above) are in place to improve accessibility and uptake. Although reported CBE uptake was similar across regional states (57% to 68%), mammography screening ranged approximately five-fold (6% to 31%) (LPPKN, 2017). The provision of subsidized mammography screening centres, particularly in rural areas, is an ongoing challenge (Mahmud and Aljunid, 2018). Providing mobile mammography screening services to women living in rural areas appeared to be targeted strategy designed to improve low screening uptake (Mahmud and Aljunid, 2018). Organising community based cancer screening camp is another strategy to improve the screening uptake especially in rural areas at low resource setting (Bashar and Aggarwal, 2020). 

A shortfall of skilled health professionals, in particular, a shortage of trained radiologists is a further challenge to providing full coverage of mammography screening services. In recognition of this problem, the current NSP-CCP has emphasised the training of professionals including breast radiologists, breast surgeons, pathologists and oncologists. Radiologists are encouraged to pursue sub-speciality training, and advanced diploma courses are offered to allied health personnel for breast imaging in order to improve manpower resources for the screening and management of breast cancer, (Ministry of Health Malaysia, 2017). 

Quality assurance and information systems are essential elements in the monitoring and evaluation of a screening programme (WHO, 2014). There is a need for standardized and consistent patient data collection to monitor screening coverage, delivery and management. The main source of data about cancer is provided by the Malaysian National Cancer Registry (NCR) which has collected patient data since 2001 (Ministry of Health Malaysia, 2008a). In 2007, the National Cancer Registry and the Regional Cancer Registries were merged under the management of the Non-Communicable Disease Sector, Ministry of Health (Ministry of Health Malaysia, 2008a). However, NCR does not collect data about utilization of cancer screening. Currently, reporting of cancer cases is voluntary (Ministry of Health Malaysia, 2016a) and there is a need to improve the completeness of cancer patients records - only 51% in the first cancer registry report (2007-2011) and 53% in the second report (2012-2016) recorded staging at diagnosis for cases (Ministry of Health Malaysia, 2016a; Ministry of Health Malaysia, 2019b). Cancer screening data are available only from public sector clinics and hospitals and the completeness of, and accessibility to, these data are variable whilst there appears to be very limited access to private sector clinic data (though data is collected and recorded for each individual patient). A plan to address these data issues was presented in the NSP-CCP in the form of a target to establish a Cancer Screening Registry and Comprehensive Cancer Data and Information System between 2014 to 2020 (Ministry of Health Malaysia, 2017). It is expected that the strategy will explore the establishment of a cancer screening registry and a cancer surveillance system that will reliable, valid and timely quality reports (Ministry of Health Malaysia, 2017). These data will aid the policy making process in terms of contributing further to the goal of achieving research-informed policy.

**Table 1:** Recommendations Regarding Breast Cancer Screening among Malaysian Women

Guideline	Breast cancer screening	General (female) population	Moderate- to High-risk (female) population	
			Risk criteria	Risk group screening recommendation
CPG Management of breast cancer (1^st^ edition; 2002)	Mammography		High-risk · Past history of breast and/or ovarian cancer · Family history of breast cancer in one or more first or second-degree relatives before the age of 50 years · History of atypia on previous breast biopsy below the age of 40 years	High-risk screening *“Annual mammogram* *in women aged 40-49 years, and annually or biennially in those 50-75 years old”*
CPG Management of breast cancer (2^nd^ edition; 2010)	Mammography	*“Biennially in women from 50 – 74 years * *(women between 40-49 years old should not be offered routinely, however, they should not be denied mammography if they desire to do)”*	High-risk · Personal history of invasive breast cancer · Lobular Carcinoma In Situ (LCIS) and Ductal Carcinoma In Situ (DCIS) · Benign breast disease with atypical hyperplasia · Ionising radiation from treatment of breast cancer, Hodgkin’s disease, etc. · Carrier of BRCA1 and 2 genetic mutation · Significant family history i.e. first-degree family with breast cancer ( MRI screening should not be performed in patients with lobular carcinoma in situ and atypical hyperplasia)	High-risk screening *“Both Mammography and MRI should be done annually at the age of 30 years. *
	Magnetic resonance imaging (MRI)			*(MRI should not be performed in patients with LCIS and atypical hyperplasia.)”*
Garis Panduan Program Pengesanan Awal Kanser Payudara Kebangsaan – [Breast Cancer Screening Programme Guidelines] (2011)	Clinical breast examination (CBE)	*3-yearly for women between 20-39 years.* *Annually for women 40 years and above. *		
	Mammogram	Criteria A: At least ONE of the following factors (Annual mammogram) · Significant family history i.e. first-degree family with breast cancer (mother, sister, daughter) · Carrier of BRCA1 and 2 genetic mutation · Atypical hyperplasia in breast biopsy Criteria B: At least TWO of the following factors (Biennial mammogram) · Never gave birth (or) gave birth to a child after 30 years of age · Early menarche (less than 12 years of age) · Late menopause (more than 55 years of age) · Receiving hormone replacement therapy (HRT) · Obesity: Body Mass Index (BMI) ≥ 27.5		
CPG Management of breast cancer (3^rd^ edition; 2019)	Clinical breast examination (CBE)	*CBE should initiate from 35 years of age. *		High-risk screening (with pathogenic variants in BRCA1, BRCA2 and PALB2) *Biennial CBE from 25 years of age.*
	Mammogram	*Biennially in women from 50 – 74 years.*	Moderate-risk · Lifetime risk: >17% but <30% lifetime risk, which may include women with pathogenic/likely pathogenic variants in PALB2 regardless of family history of breast cancer and, individuals with pathogenic/likely pathogenic variants in ATM and CHEK2 and at least one first degree relative affected by breast cancer. High-risk · Lifetime risk: >30%, which may include women with pathogenic/likely pathogenic variants in PALB2 and strong family history of breast cancer, or individuals where BOADICEA* or other risk prediction tools suggest a high-risk based on family history of breast cancer. · In local setting, high-risk criteria of developing breast cancer are * BRCA mutation * First-degree relatives of BRCA carrier who have not been tested * History of chest irradiation at young age * Personal history of breast cancer * Strong family history of breast or ovarian cancer; first- or second-degree relatives	Moderate-risk screening *Annually in women from 40-49 years. * *Annually or biennially in women 50-59 years. Biennially in women 60 years onwards.* High-risk screening (without genetic variant) *Consider mammography screening from 30-39 years.* *Annually in women 40-59 years.* * Biennially in women 60 years onwards.* High-risk screening (with pathogenic variants in BRCA1, BRCA2 and PALB2) *Annual mammogram in women 40-69 years. * *Biennial mammogram in women 70 years onwards.*
	Magnetic resonance imaging (MRI)			High-risk screening (with pathogenic variants in BRCA1, BRCA2 and PALB2) *Annual MRI in women 30-49 years. *

* BOADICEA (Breast and Ovarian Analysis of Disease Incidence and Carrier Estimation Algorithm) risk assessment tool

**Table 2:** Summary of Guidelines and Strategic Plans for Cancer Control in Malaysia

Guidelines and Strategic plans	Year	Recommendation related to cancer
Clinical Guidelines		
Clinical Practice Guidelines (CPG): Management of breast cancer	2002 (1^st^ edition)	Screening recommendations for early detection among the high-risk group and management of breast cancer patients
Clinical Practice Guidelines (CPG): Management of breast cancer	2010 (2^nd^ edition)	Screening recommendations for early detection in the general population, high-risk group, and management of breast cancer patients
Garis Panduan Program Pengesanan Awal Kanser Payudara Kebangsaan	2011	National breast cancer screening guidelines for the general population and high-risk group
Clinical Practice Guidelines (CPG): Management of breast cancer	2019 (3^rd^ edition)	Screening recommendations for early detection in the general population, risk groups, and management of breast cancer patients
National Strategic Plans for NCDs		
National Strategic Plan for Non-Communicable Disease (NSP-NCD)	2010-2014	To reduce the prevalence of NCDs (i.e. cardiovascular disease, diabetes and cancer)
National Strategic Plan for Non-Communicable Disease (NSP-NCD)	2016 – 2025	To reduce the burden of NCDs in Malaysia; including cancer
National Cancer Control Plans		
National Cancer Control Blueprint Master Plan (NCCB)	2008 - 2015	To reduce the burden of cancer by reducing mortality and morbidity, and improving quality of life among cancer patients and families
National Strategic Plan for Cancer Control Program (NSP-CCP)	2016-2020	Primary prevention, screening, and early detection, management, and cancer research

## Discussion

This paper presents the results of a review of public health policy relating to breast cancer screening in Malaysia that was undertaken in order to appraise progress and contribute to policy development regarding the enhancement of prevention and detection and the identification of opportunities for the improvement of services for Malaysian women with breast cancer. The policy review summarised key policy documents in relation to breast cancer screening and drew comparisons with WHO recommendations. The development of formal policies, strategies and guidelines in Malaysia began to occur in the early 2000s and now there appears to be an established policy and planning cycle that recognises that breast cancer is the commonest cancer and burden among women. The various policy documents focused on prevention, screening and management goals aimed at reducing the impact of cancer and evolved a common view of cancer as a NCD that was given priority policy status with the implication that efforts were to be directed towards the identification and reduction of risk factors (though details in the documents were scant). The results of the review indicated also that there were ongoing efforts to adopt an integrated or joined-up policy and planning approach (eg the overlapping and parallel development of NCD policy and cancer control policy at a national level). 

Monitoring and evaluation activities are required in order to ensure the relevancy, efficiency and effectiveness of the screening strategies and to achieve evidence-based policy making (Adrien et al., 2008). Policy plans did not include monitoring and evaluation or the use of performance indicators and criteria though it might be argued that the policy and planning cycle that appears to have emerged overtime represents a form of reviewing, appraisal, amending and development of strategies (Ministry of Health Malaysia, 2010b; Ministry of Health Malaysia, 2016b). However, it is evident from this review that policy regarding breast cancer, particularly screening, and its implementation have not been subjected to rigorous systematic evaluation.

Clinical guidelines were included in this review because arguably they represent government policy Clinical guidelines were included in this review because arguably they represent government policy and, also, guideline production was coordinated by the Malaysian MoH. and, also, guideline production was coordinated by the Malaysian MoH. The offer of mammography screening and its uptake varied among high-risk women due partly to the fact that referral and subsequent use depended upon a patient’s expressed preference or decision and a doctor’s professional discretion (CPG, 2002) (Ministry of Health Malaysia, 2002). The second edition of CPG (2010) which included screening of the general population of women and the high-risk population (Ministry of Health Malaysia, 2010a) differed in some respects to the National Breast Cancer Screening Guidelines (Ministry of Health Malaysia, 2011). For example, the CPG recommended mammography screening for women from the age of 50 whereas the NBCSG recommended conducting screening from 40 years old if at least one of the criteria in Table 1 was met. Developmental changes to the clinical guidelines appeared to draw from several sources including findings from large, population-based RCTs (Andersson et al., 1988; Tabár et al., 1999; Ministry of Health Malaysia, 2002), analysis of mammography screening (Secretariat, 2007; Ministry of Health Malaysia, 2010a), and the content of guidelines regarding the use of MRI screening for high-risk groups (Afonso, 2009; Saslow et al., 2007; Ministry of Health Malaysia, 2010a). However, guideline implementation appeared to be variable and there is a need to inform clinicians about the evidence-based nature of the findings and to encourage, perhaps incentivise, them to apply the guideline consistently in their clinics. The third edition of the CPG (2019), screening guidelines is based on the risk of developing breast cancer and recommendation is categorized according to the risk level (Ministry of Health Malaysia, 2019a). Women with pathogenic variance of breast cancer are emphasized in this edition and they should undergo intensive screening measures. Early referral (within 2 weeks) is recommended for women presenting with general warning signs and symptoms of breast cancer with aged younger than 35 years or high-risk group, and women with clinical signs of malignancy (Ministry of Health Malaysia, 2019a). Therefore, clinics should follow the updated clinical guidelines for screening of breast cancer in Malaysia. 

The policy review indicated that cancer policy in Malaysia has evolved to the point that it recognises that there is a need to take account of inequalities in access to screening. A study of the subsidised mammography screening programme reported that approximately 11% of participants did not collect their mammogram reports; there were no mechanisms for contacting and informing participants with normal findings; participants with abnormalities or malignancies were contacted though about 10% of these women were reluctant to accept further investigation; and women who did not present with lumps were likely to underestimate the seriousness of their condition and to not follow up for further investigation (Lee et al., 2017). Clearly, there is a need to improve pre-screening counselling and follow-up services post-mammogram as well as increase awareness about non-lump symptoms, the role of mammography screening and the importance of follow-up (Lee et al., 2017). In addition, denial, fear of a BC diagnosis and poor knowledge about BC treatment (Hisham and Yip, 2004; Lim et al., 2015) add to women’s reluctance to attend follow-up visits. 

Guidance about connectivity and patient flows through the cancer care system was unclear in the policies. For example, waiting time after referral for screening was not stated in clinical practice guidelines. Currently, BC diagnosis and treatment are provided mainly by 19 trained breast surgeons across eight government hospitals. A study in 6 out of 8 public hospitals found that 42% of BC patients experienced diagnosis delay (of more than one month from first presentation at a healthcare clinic to diagnosis); and more than a third of patients (35%) experienced treatment delay (of more than one month from diagnosis to initial treatment) (Mujar et al., 2018). The policy review found that the use of specified service targets has become established practice. For example, in the current NSP-CCP, the performance of BC management is measured in terms of (i) ‘Percentage of patients given appointment at a breast clinic for a suspicious breast lump/lesion within 14 working days of referral’ and (ii) ‘Percentage of patients going for definitive surgery for breast cancer within 4 weeks of the diagnosis’ (Ministry of Health Malaysia, 2017). The target proportions stated in the policy are >80% and >75%, respectively (Ministry of Health Malaysia, 2017). A referral and follow-up protocol for use by primary health services was included in the updated clinical guidelines - implementation is essential in order to improve the timeliness of BC management. In addition, there appears to be a need for policy strategies to give attention to improving the connectivity between screening programmes, referral and follow-up services and secondary or tertiary healthcare services in order to enhance treatment planning and care management for BC patients (Foot, Naylor and Imison, 2010).

*Concluding note*

The Ministry of Health, Malaysia has established and consolidated a 4- or 5-year planning cycle of cancer control policy development and review. Generally, the policy development process appeared to be top down and centrally focused though the specification of targets and goals in the policy provide an opportunity for benchmarking and quality assurance. The use of best available evidence was not explicit in the national cancer control policies. Prevention and screening particularly BC screening, have become permanent features of Malaysian cancer policy. Opportunistic screening is the current modus operandi due to limited resources and perhaps also to decision-making around the allocation of healthcare resources. Whilst policy recommends the use of mammogram for breast cancer screening, implementation remains challenging and policy needs to give more attention to key barriers such as low cancer awareness, differential levels of access to mammogram services (particularly in rural communities), an inadequate number of trained professionals and an underdeveloped comprehensive information system that is designed to afford scope to conduct rigorous monitoring and evaluation of policy targets and goals. A focus on raising awareness, increasing the accessibility of screening facilities and improving referral processes and the overall connectivity of the cancer care system are key steps to down-staging breast cancer in Malaysia.

## Author Contribution Statement

MDo and TTS conceptualised and planned the project and are the Co-PIs of the successful grant award from UK MRC-Newton Ungku Omar Fund. MNNH lead the document analysis. MDo, TTS, MDa, DS, SYL, SS and NS. IT supervised and guided the process. MNNH drafted the manuscript. TTS, MDo, and DS led the editing and refinement of the manuscript. All authors contributed to, reviewed, and approved the final manuscript.
